# Aquaporin‐4‐independent volume dynamics of astroglial endfeet during cortical spreading depression

**DOI:** 10.1002/glia.23604

**Published:** 2019-02-21

**Authors:** Brana Rosic, Didrik B. Dukefoss, Knut Sindre Åbjørsbråten, Wannan Tang, Vidar Jensen, Ole Petter Ottersen, Rune Enger, Erlend A. Nagelhus

**Affiliations:** ^1^ GliaLab and Letten Centre, Division of Physiology, Department of Molecular Medicine Institute of Basic Medical Sciences, University of Oslo Oslo Norway; ^2^ Office of the President Karolinska Institutet Stockholm Sweden; ^3^ Department of Neurology Oslo University Hospital Oslo Norway

**Keywords:** AQP4, aquaporin‐4, astrocytes, drainage, glia, glymphatic, interstitial fluid

## Abstract

Cortical spreading depression (CSD) is a slowly propagating wave of depolarization of gray matter. This phenomenon is believed to underlie the migraine aura and similar waves of depolarization may exacerbate injury in a number of neurological disease states. CSD is characterized by massive ion dyshomeostasis, cell swelling, and multiphasic blood flow changes. Recently, it was shown that CSD is associated with a closure of the paravascular space (PVS), a proposed exit route for brain interstitial fluid and solutes, including excitatory and inflammatory substances that increase in the wake of CSD. The PVS closure was hypothesized to rely on swelling of astrocytic endfeet due to their high expression of aquaporin‐4 (AQP4) water channels. We investigated whether CSD is associated with swelling of endfeet around penetrating arterioles in the cortex of living mice. Endfoot cross‐sectional area was assessed by two‐photon microscopy of mice expressing enhanced green fluorescent protein in astrocytes and related to the degree of arteriolar constriction. In anesthetized mice CSD triggered pronounced endfoot swelling that was short‐lasting and coincided with the initial arteriolar constriction. Mice lacking AQP4 displayed volume changes of similar magnitude. CSD‐induced endfoot swelling and arteriolar constriction also occurred in awake mice, albeit with faster kinetics than in anesthetized mice. We conclude that swelling of astrocytic endfeet is a robust event in CSD. The early onset and magnitude of the endfoot swelling is such that it may significantly delay perivascular drainage of interstitial solutes in neurological conditions where CSD plays a pathophysiological role.

## 1 INTRODUCTION

The hallmark of cortical spreading depression (CSD), a wave of neuronal hyperactivity followed by temporary silencing, was first described over 70 years ago by Artistides Leão during seizure experiments in rabbits (Leão, 1944). Later, CSD and CSD‐like events (for terminology see Dreier & Reiffurth, 2015; Hartings et al., 2017) were observed in models of migraine, stroke, subarachnoid hemorrhage, and traumatic brain injury (Ayata & Lauritzen, 2015; Charles & Baca, 2013). CSD is associated with a temporary breakdown of ionic gradients, profound neuronal swelling, extensive neurotransmitter release, multiphasic hemodynamic changes, and tissue hypoxia (Ayata & Lauritzen, 2015; Charles & Baca, 2013). An emerging concept is that CSD adds insult to injury by increasing the metabolic demands of the affected tissue concomitantly to diminishing blood supply. Thus, understanding CSD can provide valuable therapeutic targets for multiple neurological disorders (Lauritzen et al., 2011).

A recent study reported that the space around pial and cortical blood vessels temporarily closes during CSD (Schain, Melo‐Carrillo, Strassman, & Burstein, 2017). The authors proposed that impaired clearance of excitatory and inflammatory substances along paravascular pathways may be a facet of migraine pathophysiology. Unlike most tissues including the meninges (Aspelund et al., 2015; Louveau et al., 2015), the brain does not contain lymphatic vessels and is postulated to utilize the paravascular pathway as waste disposal route (Nedergaard, 2013). According to the “glymphatic hypothesis” (Iliff et al., 2012), which is debated (Abbott, Pizzo, Preston, Janigro, & Thorne, 2018; Asgari, de Zélicourt, & Kurtcuoglu, 2016; Bakker et al., 2016; Hladky & Barrand, 2014; Hladky & Barrand, 2017; Smith & Verkman, 2018), cerebrospinal fluid (CSF) enters the brain along penetrating arterioles, exchanges with interstitial fluid (ISF), and exits along veins. Data provided by Iliff et al. (2012) suggested that CSF‐ISF fluid exchange and solute clearance are dependent on aquaporin‐4 (AQP4) water channels, which are concentrated in perivascular astrocytic endfeet (Nielsen et al., 1997). It was originally proposed that interstitial waste products move by convective flow, both through the brain parenchyma and along the paravascular pathways (Iliff et al., 2012), possibly driven by arterial pulsations (Iliff et al., 2013). While recent evidence suggests that CSF movement through brain parenchyma occurs by diffusion, rather than by convection (Holter et al., 2017; Jin, Smith, & Verkman, 2016; Smith & Verkman, 2018; Smith, Yao, Dix, Jin, & Verkman, 2017), the convective nature of the paravascular flow has not been questioned (Abbott et al., 2018; Hladky & Barrand, 2018). Thus, the width of the paravascular space, interposed between the vascular tube and the glial endfoot sheath, must be a critical determinant of brain waste removal‐in acute conditions as well as in neurodegenerative diseases such as Alzheimer's disease.

Given that astrocytic endfeet may regulate the width of paravascular spaces and hence the turnover and composition of ISF, we chose to investigate endfoot volume dynamics in living mice subjected to CSD. Volume assessment was performed by two‐photon laser scanning microscopy of transgenic mice expressing enhanced green fluorescent protein (eGFP) in astrocytes (*Glt1*‐eGFP BAC mice, see Regan et al., 2007), a strategy successfully used before (Thrane et al., 2011). Combined with intravascular Texas Red‐conjugated dextran we studied how endfoot volume relates to CSD‐associated vascular constriction and dilation. To determine the role of AQP4 in endfoot volume dynamics the experiments were conducted on *Aqp4* ^−/−^ and *Aqp4* ^+/+^ *Glt1*‐eGFP BAC mice (Thrane et al., 2011). Furthermore, since anesthetic agents affect vascular tone (Zuurbier, Emons, & Ince, 2002) and intracellular signaling cascades implicated in glial volume regulation (Thrane et al., 2012), experiments were also carried out in awake, unanesthetized animals.

## 2 MATERIALS AND METHODS

### 2.1 Animals

Transgenic *Glt1*‐eGFP BAC mice (Regan et al., 2007) crossed with *Aqp4* ^−/−^ mice (Thrane et al., 2011) were used for the experiments. Mice were housed on a 12 hr light:12 hr dark cycle (lights on at 8 am), 1–3 mice per cage. The mice had free access to food and water. Experiments were conducted on mice at least 10 weeks old. Adequate measures were taken to minimize pain and discomfort and all experiments were carried out in accordance with the guidelines published in the European Communities Council Directive of November 24, 1986 (86/609/EEC). All procedures were approved by the Animal Use and Care Committee of the Institute of Basic Medical Sciences and the Faculty of Medicine at the University of Oslo, and the Norwegian Food Safety Authority (project numbers: FOTS 6123 and 12095).

### 2.2 Virus production and transfection

rAAVs serotype 1 and 2 were generated from plasmid construct pAAV‐*hSYN*‐jRGECO1a (Dana et al., 2016), as described previously (Tang et al., 2009), and purified by AVB Sepharose affinity chromatography (Smith, Levy, & Kotin, 2009). Following titration with real‐time PCR (rAAV titers about 1.0–6.0 × 10^12^ viral genomes/mL, TaqMan Assay, Applied Biosystems Inc.). At each injection site, approximately 150 nL of the virus mixture was injected.

### 2.3 Induction of anesthesia

Anesthesia was induced by 4% isoflurane in room air enriched with 20% pure oxygen. The mice were then transferred to a temperature‐controlled heating pad maintained at 37 °C and mounted on a nose cone flowing 2% isoflurane, in the same gas mixture as above. Buprenorphine 0.15 mg/kg was then injected intraperitoneally or subcutaneously and the mouse was left for 10 min before the surgery proceeded.

### 2.4 Procedures for imaging experiments on anesthetized mice

We performed a tracheostomy and artificially ventilated the mice with 2% isoflurane (1.5% during imaging) in room air with a small animal ventilator (SAR‐1000; CWE Inc.). The left femoral artery was then cannulated for blood gas analyses, enabling assessment and maintenance of physiological ranges of pO_2_, pCO_2_, and pH (pO_2_ = 80–120 mmHg, pCO_2_ = 25–35 mmHg, and pH = 7.30–7.50) by adjustment of the ventilator settings. The intra‐arterial cannula was also used to inject Texas Red‐labeled dextran (Thermo Fisher Scientific) for visualization of the vasculature.

We then created a craniotomy (3 mm in diameter) centered 2 mm posterior to the Bregma and 2.5 mm lateral of the midline, as previously described (Enger et al., 2015). A dental drill was used to carefully carve a circular furrow in the skull with intermittent application of saline for cooling and removal of debris, until the bone thickness was approximately 0.1 mm. Subsequently, the skull was soaked in saline for 10 minutes to soften before the bone flap was lifted off and dura mater was removed with a vessel dilator. Agarose (0.8%) in saline was applied on the brain surface and a coverslip was added. We placed the coverslip slightly off‐center to enable insertion of an extracellular electrode in the uncovered area. A second, smaller craniotomy was created approximately 4 mm rostral to the imaging window to enable induction of CSD waves by epidural application of KCl (3 μL, 1 mol/L).

### 2.5 Procedures for imaging experiments on awake mice

Anesthesia was maintained by 1–1.5% isoflurane in room air enriched with 20% pure oxygen. The surgical area was sterilized with iodine. The skull was exposed and cleaned. Groves were cut by scalpel in a checkboard pattern into the periosteum to enable strong adhesion of the cyanoacrylate glue that was subsequently applied. We then attached a custom‐made titanium head‐bar at the caudal end of the exposed skull and made a 2.5 mm diameter craniotomy as described above, located 3 mm posterior to the Bregma and 2.5 mm lateral of the midline.

Recombinant adeno‐associated virus (AAV) with rAAV2/1‐*hSYN*‐jRGECO1a was injected (150 nL, 20 nL/min) at three injection sites at about 400 μm depth. Care was taken to avoid the vasculature. Subsequently, a glass plug made of two coverslips of 2.5 mm and 3.5 mm, respectively, was glued together by ultraviolet light curing glue. The window was then placed so that the smaller glass coverslip rested on the cortex, while the larger coverslip rested on the bone edge around the imaging window (Huber et al., 2012).

In a subset of experiments, two noninsulated silver wire electrodes (200 μm diameter) for acquiring electrocorticogram (ECoG) during CSD, were placed on each side of the midline, right above the dura mater through small craniotomies (Enger et al., 2017). The rest of the wires were insulated with glue and dental cement and soldered to gold pins that subsequently were fastened on top of the titanium head‐bar.

A second smaller craniotomy was made approximately 4 mm rostral for the imaging window to allow induction of CSD with 2 μL of 1 mol/L KCl or by pinprick. We temporarily sealed the rostral window with KWIK‐SIL (World Precision Instruments). Finally, dental cement was applied to all parts of the exposed skull. The mice were given buprenorphine 0.15 mg/kg for 2 days after surgery.

### 2.6 Two‐photon microscopy

For experiments on anesthetized mice eGFP and Texas Red fluorescence was recorded by a two‐photon laser scanning microscope model Ultima from Bruker/Prairie Technologies. Images were recorded from 50 to 100 μm depth, with an Olympus 25×, 1.05 NA water‐immersion objective (XLPLN 25 WMP 1.05 NA), using 900 nm laser pulses for excitation with an average power of 6–14 mW, as measured by LaserCheck (Coherent). The laser was a model “Mai Tai DeepSee” (Spectra Physics). Time‐series and electrophysiological recordings were triggered by pCLAMP 10.4 (Molecular Devices, LLC) to enable precise timing of the DC shifts and corresponding images. Time‐series were performed with frame rates of 1–4 Hz.

For experiments on awake mice jRGECO1a, eGFP, or Texas Red fluorescence was recorded by two‐photon laser scanning microscopy on the same system as described in Enger et al., 2017. The microscope objective was a Nikon 16 × 0.8 NA water‐immersion objective (model CFI75 LWD 16XW; Tokyo, Japan). The photomultiplier tubes are Peltier cooled units (model 7422PA‐40, Hamamatsu Photonics K.K.). An excitation wavelength of 900 nm was used and images were sampled at 256 × 256 pixels of a field‐of‐view of approximately 50 × 50 μm at 50–120 μm below the pial surface (cortical layers 1 and 2/3) with frame rates of 3–6 Hz. The first imaging session was performed at least 10 days after implantation of the chronic imaging window. Mice were then trained for 2–3 days with handling and getting used to head fixation on a spherical treadmill or rotating disk before data collection commenced. The interval between CSD‐induction experiments was minimum 12 hrs.

### 2.7 Image analysis

Time‐series of imaging data were imported into Fiji ImageJ (Schindelin et al., 2012), and regions of interest (ROIs) were manually selected based on morphology using either a circular selection or Cell Wand Tool to label cross‐sectional areas of arterioles and astrocytic endfeet, respectively. The videos were preprocessed with a light Gaussian blurring filter and contrast normalization (ImageJ function “enhance contrast”). Subsequently, endfoot and vessel cross‐sectional areas were detected based on manually set thresholds within the defined ROIs. The thresholded areas were monitored throughout the time series to ensure correct area estimates. As the aim was to determine how areas change over time in individual vessels it was not considered necessary to blind the experimenter to genotype. The collected data was then analyzed by custom‐written MATLAB scripts (R2016b, MathWorks, Inc.).

### 2.8 Statistics

Statistical analyses were performed using Microsoft Excel. Unless stated otherwise, all values are given as mean ± standard error of the mean (*SEM*). The analyses were tested with a one‐tailed Student's *t*‐test when comparing cross sectional area in the same specimen, otherwise we performed a two‐tailed Student's *t*‐test.

## 3 RESULTS

**Figure 1:**
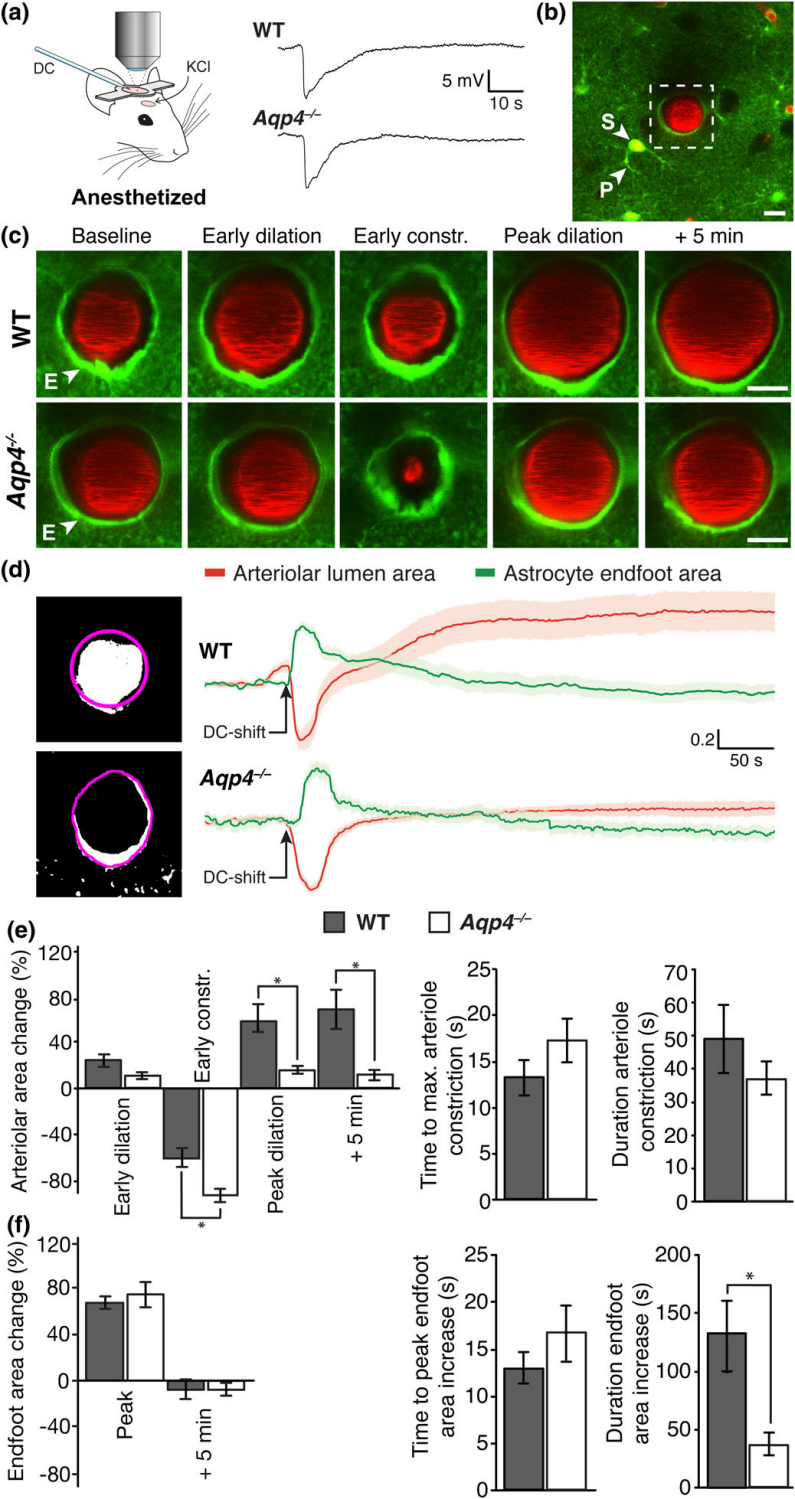
CSD is associated with changes in arteriolar tone and swelling of astrocytic endfeet in anesthetized *Glt1*‐eGFP BAC transgenic mice. (a) Experimental setup. A mouse with two cranial windows was positioned under the objective. The caudal craniotomy overlying the visual cortex was the two‐photon imaging window. CSD was elicited through a rostral window by KCl or pinprick. Example traces of DC‐shifts in *Aqp4* ^*+/+*^ (WT) and *Aqp4* ^*−/−*^ *Glt1*‐eGFP BAC transgenic mice. (b) Two‐photon image of an *Aqp4* ^*−/−*^ *Glt1*‐eGFP BAC mouse showing astrocytes in green and blood vessels in red. The vessels were outlined with intravascular Texas red‐conjugated dextran. Boxed area shows a penetrating arteriole and its astrocytic endfeet displayed at higher magnification in c. (c) Example two‐photon images of WT and *Aqp4* ^*−*/−^ *Glt1*‐eGFP BAC transgenic mice exposed to CSD. An arteriole and its endfeet were recorded in a cross‐section plane 50–100 μm below the cortical surface, avoiding vessel segments including astrocytic somata. Images shown are taken at baseline, peak early arteriolar dilatation and constriction, and 5 min after maximum arteriolar constriction. (d) Left panel, binary images obtained after fluorescence thresholding showing the arteriolar lumen (upper image) and periarteriolar astrocytic endfeet (lower image). Such images were used for quantification of cross‐sectional area. Regions of interests are shown in magenta. Right panel, mean traces representing normalized cross‐sectional area of arteriolar lumen (red) with SEM (light red) and astrocytic endfeet (green) with SEM (light green) in WT (n = 10 recordings in 8 mice) and *Aqp4* ^*−*/−^ (n = 8 recordings in 7 mice) mice. (e) Bar diagram of the arteriolar lumen cross‐sectional area changes in WT and *Aqp4* ^*−/−*^ *Glt1*‐eGFP BAC mice. (f) Bar diagram of the endfoot cross‐sectional area changes. Values are mean ± SEM. Asterisks indicate *p* < 0.05 for comparison. Scale bars: 10 μm [Color figure can be viewed at wileyonlinelibrary.com]

CSD was elicited in anesthetized mice by focal epidural application of KCl through a rostral craniotomy while the visual cortex was imaged by two‐photon microscopy through a separate craniotomy (Figure 1a). A pronounced deflection of the DC potential—the hallmark of CSD—was seen in both *Aqp4* ^+/+^ (WT) and *Aqp4* ^−/−^ *Glt1*‐eGFP BAC mice. Astrocytic somata, processes and endfeet were easily identified by their green fluorescence and typical morphology (Figure 1b,c). Cerebral blood vessels were outlined by intravascular Texas Red‐labeled dextran. We zoomed in on penetrating cortical arterioles, identified by their morphology and direction of blood flow, and the surrounding astrocytic endfeet 50–100 μm below the pial surface (Figure 1c). The cross‐sectional area of endfeet and the arteriolar lumen was used as a proxy for endfoot volume and arteriolar tone, respectively.

In both WT and *Aqp4* ^−/−^ mice, CSD induced multiphasic changes in cross‐sectional area of the arteriolar lumen (Figure 1c,e), as described previously for WT mice (Enger et al., 2015). The peak arteriolar constriction was more prominent in *Aqp4* ^*−/−*^ mice (85.0 ± 3.1% reduction of arteriolar lumen area, n = 8 waves in seven mice, *p* < 0.001 for comparison with baseline) than in WT mice (61.4 ± 8.0% reduction, n = 10 waves in eight mice, *p* < 0.001 for comparison with baseline; *p* = 0.025 for comparison between genotypes; Figure 1d,e), albeit the duration of the constriction did not differ between the genotypes (*p* = 0.36). Also the following vasodilatation was strikingly smaller in AQP4 deficient mice, with peak increase of arteriolar lumen area of only 15.8 ± 3.5% (*p* < 0.001 for comparison with baseline) compared with 62.6 ± 15.2% in WT mice (*p* < 0.001 for comparison with baseline; *p* = 0.017 for comparison between genotypes, Figure 1d,e). Five minutes after the DC deflection the arteriolar lumen area increase was 11.9 ± 5.9% in mutants and 69.1 ± 16.4% in WT (*p* = 0.009 for comparison between genotypes, Figure 1d,e).

In both WT and *Aqp4* ^−/−^ mice astrocytic endfeet showed bi‐phasic changes in cross‐sectional area during CSD (Figure 1c,d). A rapid increase and decrease was followed by a delayed recovery toward baseline values. In WT mice the immediate arteriolar constriction was associated with a 66.7 ± 5.5% peak increase in endfoot cross‐sectional area (n = 10 waves, in eight mice, *p* < 0.001 for comparison with baseline; Figure 1f). The peak endfoot swelling coincided with maximum arteriolar constriction (Figure 1d). Time to peak endfoot cross‐sectional area and time to maximum arteriolar constriction were 12.7 ± 1.6 s and 13.3 ± 1.8 s, respectively, which was not significantly different (*p* = 0.82, Figure 1e,f). The duration of endfoot swelling and arteriolar constriction, defined as the width at 20% of peak values, were 132.6 ± 35.8 s and 49.1 ± 4.0 s, respectively (*p* = 0.058 for comparison, Figure 1e,f).

Knockout of AQP4 minimally affected the CSD‐associated changes in endfoot cross‐sectional area in anesthetized mice (Figure 1c,d). During the initial vasoconstriction, endfoot cross‐sectional area of *Aqp4* ^−/−^ mice increased to 75.2 ± 10% (n = 8 waves in seven mice, *p* < 0.001 for comparison with baseline, Figure 1f). However, neither peak values (*p* = 0.51) nor time to peak (16.8 ± 2.7 s in mutants, *p* = 0.26; Figure 1f) differed between the two genotypes. In contrast, the duration of the endfoot cross‐sectional area increase was significantly shorter in mutants (41.9 ± 7.2 s) than in WT (*p* = 0.04, Figure 1f), even though the duration of the arteriolar constriction did not differ between the genotypes (see above).

**Figure 2:**
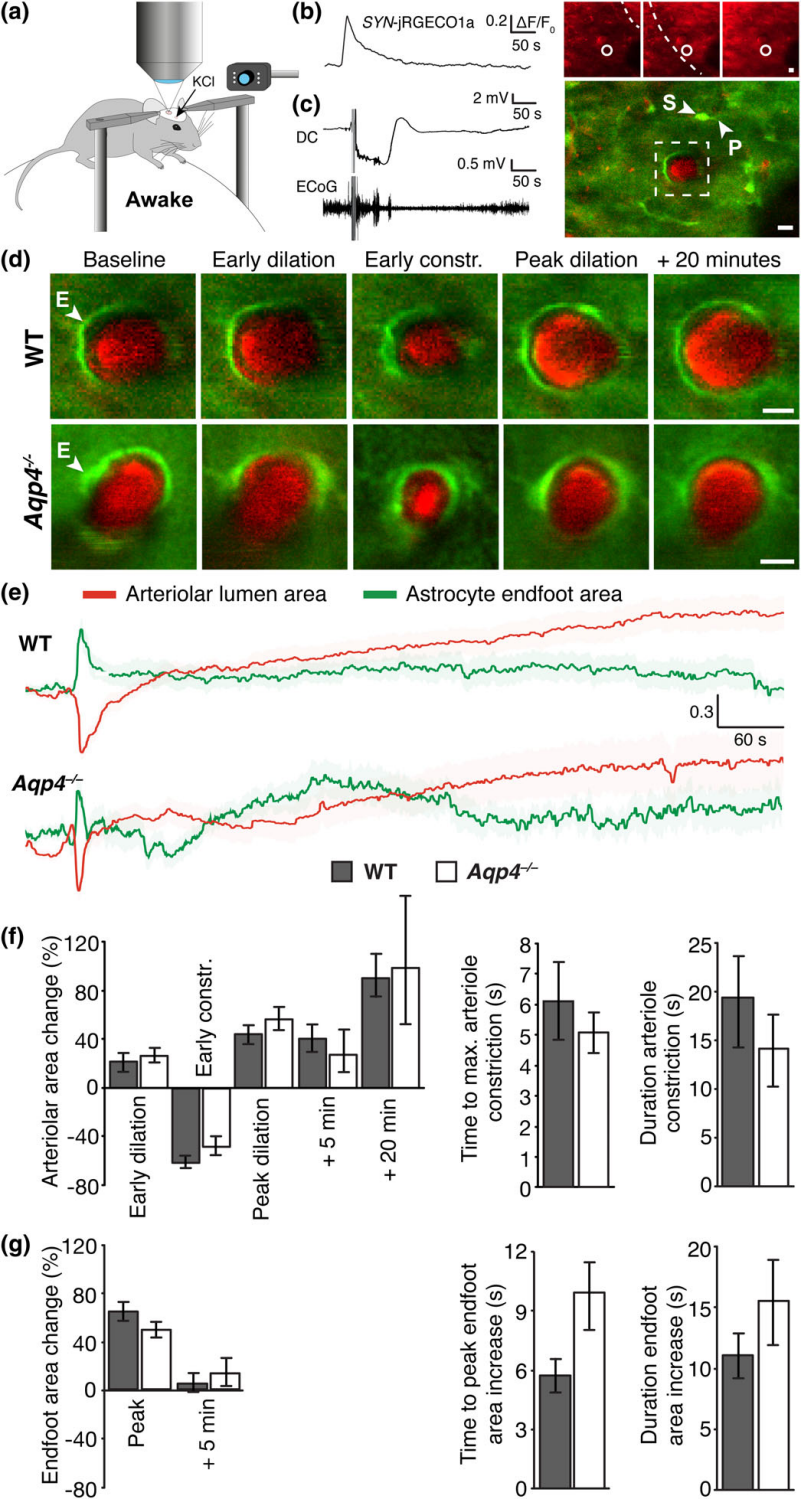
CSD is associated with changes in arteriolar tone and swelling of astrocytic endfeet in awake *Glt1*‐eGFP BAC transgenic mice. (a) Experimental setup. A head‐fixed awake mouse was positioned on a spherical trackball under the two‐photon microscope. CSD was elicited by KCl application or pinprick through the rostral craniotomy while imaging was performed through the caudal craniotomy. An infrared camera monitored mouse behavior. (b) Trace of *hSYN*‐jRGECO1a fluorescence from the circular region of interest in the snap‐shot fluorescence images to the right. Passage of a CSD wave was associated with a steep increase in neuronal jRGECO1a fluorescence. Stippled line in images indicates the CSD wavefront. (c) Example traces of DC potential and ECoG (obtained in a subset of the animals). Right panel shows a two‐photon image of an awake WT *Glt1*‐eGFP BAC transgenic mouse with *hSYN*‐jRGECO1a and intravascular Texas red‐labeled dextran. Boxed region is shown at higher magnification in upper panel of d. (d) Snap‐shot images of an arteriole and its endfeet in WT (upper panel) and *Aqp4* ^*−*/−^ (lower panel) *Glt1*‐eGFP BAC transgenic mice during CSD. The sequence of images is as in Figure 1, except the last image which was taken 20 min after maximum arteriolar contraction. Arrowheads denote astrocytic soma (S), process (P), and endfeet (E). (e) Mean traces representing normalized cross‐sectional area of arteriolar lumen (red) with SEM (light red) and astrocytic endfeet (green) with SEM (light green) in WT (arteriolar lumen: N = 13 recordings in six mice; endfoot: N = 12 recordings in eight mice) and *Aqp4* ^*−*/−^ (arteriolar lumen: N = 12 recordings in seven mice; endfoot: N = 11 recordings in five mice). (f) Bar diagram of the cross‐sectional area changes of arteriolar lumina. (g) Bar diagram of the cross‐sectional area changes of astrocytic endfeet. Values are mean ± *SEM*. Scale bars: 10 μm [Color figure can be viewed at wileyonlinelibrary.com]

Given the effects of anesthetic agents on vascular tone and signaling cascades implicated in astrocyte volume regulation (see Section 1) we also assessed arteriolar constriction and endfoot volume during CSD in fully awake, head‐fixed mice positioned on a spherical treadmill under the objective (Figure 2a). CSD was elicited by KCl or pinprick 2–6 weeks after preparation of a chronic cranial window and intracortical injection of virus with the construct for the red fluorescent Ca^2+^ sensor jRGECO1a. The *hSYN* promoter was used to drive jRGECO1a expression in neurons. An infrared camera enabled us to monitor mouse behavior during the experiments. As previously reported, CSD elicitation was not associated with any noticeable discomfort (Akcali, Sayin, Sara, & Bolay, 2010; Koroleva & Bures, 1993). Successful triggering of CSD in awake mice was verified by passage of neuronal Ca^2+^ waves (Enger et al., 2017), detected with *hSYN*‐jRGECO1a (Figure 2b). Using this approach we avoided having electrodes inserted in the unanesthetized mice during the imaging session. Only in a subset of awake mice, DC potential and ECoG changes were recorded and confirmed the negative DC‐shift and inhibition of neural activity characteristic for CSD (Figure 2c). As in anesthetized mice we used Texas Red‐labeled dextran to visualize the vasculature.

As in anesthetized mice, CSD in awake mice was associated with multiphasic changes in arteriolar lumen cross‐sectional area (Figure 2d,e). The peak amplitude of the early arteriolar dilation and constriction (22.1 ± 7.0% and 62.2 ± 4.5%, respectively, n = 13 waves in six mice, Figure 2f) in awake WT mice mimicked those of anesthetized WT mice. However, the duration of the early arteriolar constriction was considerably shorter in awake (19.4 ± 4.3 s, Figure 2f) than in anesthetized mice (*p* = 0.026). The arteriolar dilation following the initial constriction persisted throughout the image session (over 1 hr). While this dilation had a down sloping trend, it was still 91.3 ± 21.5% (n = 9 waves in four mice) increased relative to baseline value 20 min after passage of the neuronal Ca^2+^ wavefront.

Awake *Aqp4* ^−/−^ mice did not differ from awake WTs in CSD‐associated changes in arteriolar lumen cross‐sectional area (early dilation, early constriction, peak dilation, and dilation 5 min after the DC deflection in mutants were 26.8 ± 5.8%, 48.3 ± 6.4%, 56.7 ± 12.1%, and 27.9 ± 17.1%, respectively), time to peak arteriole constriction (5.1 ± 0.7 s in mutants) or duration of arteriole constriction (14.1 ± 3.9 s in mutants; Figure 2f).

CSD was associated with an increase in endfoot cross‐sectional area also in awake WT mice (64.7 ± 6.9% increase from baseline, n = 12 waves in eight mice, *p* < 0.001, Figure 2d,e,g). The peak increase did not differ between awake and anesthetized WT animals (*p* = 0.83). However, both time to peak (5.7 ± 0.8 s) and duration (11 ± 1.9 s) of the endfoot cross‐sectional area increase was noticeably shorter in awake versus anesthetized WT mice (time to peak: *p* = 0.002, duration: *p* = 0.01). CSD‐associated changes in endfoot cross‐sectional area in awake *Aqp4* ^−/−^ mice did not differ from those in awake WT mice (increase from baseline, time to peak and duration of the increase in mutants were 50.3 ± 4.7%, 9.9 ± 2.6 s and 15.5 ± 3.6 s, respectively, n = 11 waves in five mice, Figure 2g).

## 4 DISCUSSION

The main finding of this study is that CSD is associated with AQP4‐independent swelling of astrocytic endfeet around penetrating arterioles. To our knowledge, swelling of astrocytic endfeet has never before been quantified in living mice, although it was observed in CSD by Risher, Croom and Kirov (2012). The CSD‐associated endfoot swelling coincided with the initial arteriolar constriction but had a more delayed recovery, typically approaching baseline values within 2–3 min.

Swelling of endfeet in CSD was recently hypothesized to underlie closure of paravascular spaces and impairment of glymphatic flow (Schain et al., 2017). The authors showed that the space around penetrating arterioles closed in parallel with the initial CSD‐induced arteriolar constriction, as reflected by similar time to peak values. Our data indeed point to a robust endfoot swelling, but indicate that this swelling is short‐lasting compared with the duration of the paraarteriolar space closure. Specifically, Schain et al. found that paraarteriolar space closure was substantial (~50% of baseline value) even 10 min after the onset of vasoconstriction, whereas our data indicated that endfoot volume recovered within 2–3 min (see Figure 1d,f). Thus, compression by swollen endfeet is a mechanism that can explain the early phase of paraarteriolar space closure, but not the late phase.

The pronounced swelling of astrocytic endfeet that occurs in CSD is likely to reflect a collapse of endfoot function that may well explain the late phase of paraarteriolar space closure. We have shown previously that astrocytic swelling is associated with brisk Ca^2+^ signaling and hypothesized that abnormal Ca^2+^ signaling might activate proteases that in turn lead to disruption of the molecular assemblies that are critically involved in endfoot function (Szokol et al., 2015). Persistent endfoot pathology will compromise homeostatic functions at the brain–blood interface and could have long lasting effect on paraarteriolar space volume.

Anesthetized *Aqp4* ^−/−^ and WT mice did not differ in the magnitude of endfoot swelling, but the recovery phase was faster in the knockouts. The minimal effect of *Aqp4* deletion on endfoot swelling during CSD indicates that the excessive water influx during CSD occur through membrane proteins other than aquaporins, for example, through chloride cotransporters as demonstrated in pyramidal neurons (Steffensen, Sword, Croom, Kirov, & MacAulay, 2015). In line with this idea AQP4‐independent swelling of astroglial somata was observed during peri‐infarct depolarizations (Rakers, Schmid, & Petzold, 2017). Recent studies point to a high complexity when it comes to AQP4 mediated water transport and its dependence or independence on other membrane proteins (Benfenati et al., 2011; Jo et al., 2015; Mola et al., 2016; Rakers et al., 2017; Toft‐Bertelsen, Krizaj, & MacAulay, 2017).

It is well known that anesthetic agents, including isoflurane which was used in our study, perturb vascular tone (Zuurbier et al., 2002) and astrocytic Ca^2+^ signaling (Thrane et al., 2012), both of which could affect endfoot volume regulation. We, therefore, extended our experiments to include unanesthetized mice, using genetically encoded Ca^2+^ sensors in neurons to confirm passage of the CSD wave (Enger et al., 2017). Swelling of endfeet also occurred during CSD in the fully awake state, with peak cross‐sectional area increase similar to that found during anesthesia. The dynamics of the endfoot swelling was, however, faster in awake animals, as was the dynamics of the early arteriolar constriction. These experiments confirmed that endfoot swelling is a robust feature of CSD in living animals, only moderately affected by anesthesia. In awake animals neither arteriolar constriction nor endfoot swelling depended on AQP4, indicating that the differences between anesthetized WT and *Aqp4* ^−/−^ mice were secondary to anesthesia.

We conclude that astrocyte endfeet surrounding penetrating arterioles swell temporarily and robustly during CSD, both in anesthetized and awake mice. The endfoot swelling is not mediated by AQP4 but coincides with vascular constriction. We propose that endfoot swelling contributes to paravascular space closure in the early phase of CSD through a direct mechanical compression of the paraarteriolar space. In the latter phase of CSD, the closure of the paraarteriolar space may reflect a breakdown of the homeostatic processes that depend on normal endfoot function.
